# Human protein C concentrate to restore physiological values in adult septic shock patients: effects on microcirculation

**DOI:** 10.1186/cc10808

**Published:** 2012-03-20

**Authors:** A Morelli, A Donati, A Di Russo, F D'Ippolito, C Raffone, A D'Egidio, MR Lombrano, S Tondi, E Damiani, V Cecchini, A Orecchioni, P Pietropaoli

**Affiliations:** 1 University La Sapienza, Rome, Italy; 2 Marche Polytechnique University, Ancona, Italy

## Introduction

We investigated whether human protein C (PC) concentrate to restore physiological values in adult septic shock patients can influence microcirculatory blood flow.

## Methods

We enrolled 36 septic shock patients with plasma protein C activity <60%. Patients were randomly allocated to be treated with either a continuous infusion of PC concentrate at 3 UI/kg/hour for 72 hours to reach plasma protein C activity between 70 and 120% or a standard treatment (control; each *n *= 18). In both groups, NE was titrated to achieve a MAP between 65 and 75 mmHg. Data from right heart catheterization and sidestream dark-field imaging were obtained at baseline and after 24, 48 and 72 hours.

## Results

For the same MAP and cardiac output, no significant differences were found between groups in terms of microvascular flow index of the small vessels (MFIs) and perfused vessel density (PVD). Results are summarized in Table 1.

**Table 1:** Microcirculatory variables

	Baseline	24 hours	48 hours	72 hours
MFIs				
Treated	2.8(2.6; 3)	3 (2.7; 3)	2.9 (2.8; 3)	3 (2.9; 3)
Controls	2.8 (2.1; 2.9)	2.8 (2.1; 2.8)	2.8 (2.2; 3)	3 (2.6; 3)
PVD				
Treated	17.8 (16.5; 22.2)	19.7 (17.4; 22.5)	19.7 (18.1; 23)	19.9 (17; 22.2)
Controls	20.2 (17.4; 23.5)	18.8 (17.6; 20.2)	19.4 (17.5; 20.7)	18.7 (17.5; 21.2)

## Conclusion

The administration of human PC concentrate did not influence microcirculatory blood flow in septic shock patients.

